# Mitochondrial Quality Control Processes at the Crossroads of Cell Death and Survival: Mechanisms and Signaling Pathways

**DOI:** 10.3390/ijms25137305

**Published:** 2024-07-03

**Authors:** Emanuele Marzetti, Riccardo Calvani, Francesco Landi, Helio José Coelho-Júnior, Anna Picca

**Affiliations:** 1 Fondazione Policlinico Universitario “Agostino Gemelli” IRCCS, L.go A. Gemelli 8, 00168 Rome, Italy; emanuele.marzetti@policlinicogemelli.it (E.M.); riccardo.calvani@unicatt.it (R.C.); francesco.landi@unicatt.it (F.L.); coelhojunior@hotmail.com.br (H.J.C.-J.); 2 Department of Geriatrics, Orthopedics and Rheumatology, Università Cattolica del Sacro Cuore, L.go F. Vito 1, 00618 Rome, Italy; 3 Department of Medicine and Surgery, LUM University, SS100 km 18, 70010 Casamassima, Italy

**Keywords:** cell death, DAMPs, extracellular vesicles, inflammaging, interleukin, mtDNA, mitochondrial-derived vesicles, mitochondrial quality control, pyroptosis, SASP

## Abstract

Biological aging results from an accumulation of damage in the face of reduced resilience. One major driver of aging is cell senescence, a state in which cells remain viable but lose their proliferative capacity, undergo metabolic alterations, and become resistant to apoptosis. This is accompanied by complex cellular changes that enable the development of a senescence-associated secretory phenotype (SASP). Mitochondria, organelles involved in energy provision and activities essential for regulating cell survival and death, are negatively impacted by aging. The age-associated decline in mitochondrial function is also accompanied by the development of chronic low-grade sterile inflammation. The latter shares some features and mediators with the SASP. Indeed, the unloading of damage-associated molecular patterns (DAMPs) at the extracellular level can trigger sterile inflammatory responses and mitochondria can contribute to the generation of DAMPs with pro-inflammatory properties. The extrusion of mitochondrial DNA (mtDNA) via mitochondrial outer membrane permeabilization under an apoptotic stress triggers senescence programs. Additional pathways can contribute to sterile inflammation. For instance, pyroptosis is a caspase-dependent inducer of systemic inflammation, which is also elicited by mtDNA release and contributes to aging. Herein, we overview the molecular mechanisms that may link mitochondrial dyshomeostasis, pyroptosis, sterile inflammation, and senescence and discuss how these contribute to aging and could be exploited as molecular targets for alleviating the cell damage burden and achieving healthy longevity.

## 1. Introduction

Aging may be viewed as the outcome of a constant “duel” between resilience and the accumulation of damage [1]. High resilience at a young age can completely offset damage accrual. However, an accumulation of damage over time that is not fully compensated for by resilience leads to an imbalance and a predisposition to a phenotype identified as frailty [1]. Resilience becomes overwhelmed toward the end of life, when even light stressors can cause a rapid and unopposed accumulation of damage that rapidly leads to frailty and eventually death [1].

At the cellular level, extrinsic and intrinsic insults, including but not limited to oxidative and genotoxic stress, epigenetic changes, chromatin disorganization, altered proteostasis, mitochondrial dysfunction, chronic inflammation, and nutrient deprivation that overcome the resilience potential can trigger cell senescence [2]. The latter is a state in which cells remain viable but lose their proliferative capacity, undergo metabolic alterations, and become resistant to apoptosis [3,4,5,6,7,8]. This is accompanied by complex cellular changes (e.g., nuclear and vacuolar enlargement, plasma membrane alterations) that arrest growth and enable the development of a senescence-associated secretory phenotype (SASP) [9,10].

Mitochondria are cellular organelles involved in energy provision, biosynthesis of macromolecules (i.e., amino acids, lipids, and nucleotides), calcium buffering, and heme and iron–sulfur cluster biosynthesis. All these activities are essential for regulating cell survival and death (i.e., apoptosis), and they are negatively impacted by aging. A decline in mitochondrial signaling is observed during aging, which is also accompanied by the development of a chronic state of low-grade sterile inflammation [11,12]. Of note, age-associated sterile inflammation shares some features and mediators with SASP [13]. Indeed, the unloading of damage-associated molecular patterns (DAMPs) at the extracellular level triggers sterile inflammatory responses. Mitochondria contribute to the generation of DAMPs with proinflammatory molecules [11]. Mitochondrial constituents, including mitochondrial DNA (mtDNA), can be released through different routes. The displacement of mtDNA via mitochondrial outer membrane permeabilization (MOMP) under an apoptotic stress has recently been shown to trigger senescence programs [14].

Other pathways, such as those of programmed cell death, can contribute to sterile inflammation. Pyroptosis is a caspase-dependent inducer of systemic inflammation, which is also elicited by mtDNA release and contributes to aging [15], although not all cell-free mtDNA has proinflammatory features [16]. However, the relationship among mitochondrial signaling, sterile inflammation, pyroptosis, and senescence in the development of age-related conditions warrants investigation.

Herein, we overview the cellular pathways and molecular mechanisms that may link mitochondrial dyshomeostasis, pyroptosis, sterile inflammation, and senescence. We also discuss how these pathways contribute to aging and may eventually be exploited as biological targets for alleviating the burden of cell damage and achieving healthy longevity.

## 2. Mitochondrial Quality Control Mechanisms and Aging

Altered mitochondrial signaling and reduced resilience to stress are features of aging. The set of processes comprising mitochondrial biogenesis, dynamics, mitophagy, and the unfolded protein response that are in place to maintain mitochondrial quality are negatively impacted by the aging process [17].

Mitochondrial biogenesis involves the generation of novel organelles and is orchestrated by a tight coordination of the activity of nuclear and mitochondrial transcription factors (i.e., nuclear respiratory factors, peroxisome proliferator-activated receptor gamma coactivator 1-alpha, and the mitochondrial transcription factor A) under the regulatory control of energy sensors (i.e., AMP-activated protein kinase (AMPK)) [18,19]. As an outcome of mitochondrial biogenesis stimulation, mtDNA replication and transcription are triggered. Indeed, variations in the mtDNA content are indicators of mitochondrial biogenesis [20]. However, conflicting results have been reported on the extent of age-associated changes in mitochondrial biogenesis, in both preclinical models and humans. While several studies reported an age-associated increase in the mtDNA content in the skeletal muscle [21,22], investigations using electron microscopy analysis did not show changes in the number of skeletal muscle mitochondria [23,24]. Furthermore, studies in rodents described specific age-associated declines in the mtDNA content across tissues (i.e., brain, heart, liver, and skeletal muscle) [25,26,27]. These findings were in line with results showing reduced mitochondria bioenergetics and activity of mitochondrial enzymes in the skeletal muscle [28,29] and brain of old rats [30]. Regardless of these discrepancies, likely due to different methodological approaches, the heterogeneity of the skeletal muscle mitochondrial structure and function indicates that, besides the mitochondrial number and content, their functional state may be more relevant and worth being assessed more comprehensively. As previously mentioned, the maintenance of mitochondrial function relies on several processes, including mitochondrial dynamics, biogenesis and mitophagy, collectively indicated as mitochondrial quality control (MQC) mechanisms.

Mitochondrial dynamics, the set of processes that regulate mitochondrial fusion and fission, are crucial readouts of mitochondrial plasticity. A decline in the expression of proteins involved in mitochondrial fission (e.g., dynamin-related protein 1 (DRP1) and fission 1 (FIS1)) has been described with age and gene manipulations to restore their expression improve mitochondrial function and morphology. Of note, deletion of the fission protein DRP1 orthologue in yeasts significantly delays aging, thus implicating mitochondrial fission in the regulation of the organismal lifespan [31]. Conversely, a higher mitochondrial fission was identified in diabetic mice and associated with impaired insulin signaling and skeletal muscle mitochondrial dysfunction [32]. As for mitochondrial fusion, different interventions eliciting pro-longevity pathways in *C. elegans* have been associated with increased mitochondrial fusion [33]. In addition, studies in *Drosophila* have shown a shift toward increased mitochondrial elongation that was associated with an increase in the fusion protein mitofusin (MFN) in midlife [34]. In keeping with these findings is the identification of a shift toward fusion in mitochondria from the skeletal muscle of very old adults with hip fracture [35]. Altogether, these observations indicate that mitochondrial fusion may serve as a conserved mechanism that preserves mitochondrial structure and function and may be associated with improved longevity. However, whether promoting fusion is a strategy for coping with age-associated mitochondrial dysfunction needs to be established. Of note, neuronal overexpression of the mitophagy E3 ubiquitin ligase Parkin in aged flies was shown to impact mitochondrial dynamics, blunt proteotoxicity, and ameliorate mitochondrial function [36]. An extension of both mean and maximum lifespan was also achieved in Parkin-overexpressing flies [36]. Such findings, while supporting the relevance of these processes to longevity, also highlight the interconnected nature of mitochondrial dynamics and mitophagy and the need to consider them in the integrated view of MQC processes.

Mitophagy is a specific form of autophagy that degrades and recycles depolarized mitochondria upon tagging [37]. Mitophagy has gained increased attention as a hallmark of aging [38] and health [39]. Several mitophagy pathways have been evolutionary selected such that mitophagy can be distinguished into PTEN-induced kinase 1 (PINK1)–Parkin dependent and PINK1–Parkin independent [37]. Regardless of the operating route, a dysfunctional mitophagy leads to the accrual of inefficient mitochondria and has been implicated in aging and chronic diseases [40]. During early life, programmed mitophagy supports cell development [41,42], whereas basal mitophagy over the life course allows damaged organelles to be continuously recycled [43,44]. Furthermore, mitophagy can be triggered acutely by internal and external stimuli that challenge mitochondrial integrity and function [45].

Finally, mitochondrial quality relies on the activity of mitochondrial proteases and peptidases that supervise protein maturation (e.g., correct import and folding) and assist in the degradation of defective and/or redundant proteins [46,47,48]. This quality control process is enacted by the mitochondrial unfolded protein response (mtUPR) that senses cell protein aggregates and triggers protective responses [49]. As part of the mtUPR, the cell actively transcribes nuclear genes coding for mitochondrial chaperones and proteases that support the removal of toxic protein aggregates and alleviate the mitochondrial protein misfolding burden [50,51,52]. Recent studies have revealed that a mitochondrial-associated degradation pathway operates via the proteasome and mediates ubiquitination and recycling of proteins of the mitochondrial matrix and of the inner and outer mitochondrial membranes. This contributes to preserving overall mitochondrial function in the setting of mild oxidative stress [53]. An altered protein-folding capacity has been associated with the accrual of highly folded or nondegradable protein aggregates that can perturb cell proteostasis. Altered proteostasis together with mitochondrial dysfunction have been implicated in aging and associated diseases, especially neurodegeneration [52,54,55]. However, several open questions remain that hamper the development of therapeutic strategies and are related to the exact definition of the system response, stress induction, and the identity of the signals, as well as the need for reliable readouts of such a complex process [56].

As discussed in the next sections, all the processes implicated in MQC and signaling are connected in a network with other features of aging via general mechanisms, such as inflammation and oxidative stress. Declines in any of these processes may result in a failure of cell homeostasis that leads to a loss of resilience and the development of frailty.

## 3. Mitochondrial Dysfunction and Inflammation: Culprits or Bystanders?

Aging cells acquire ultrastructural and functional characteristics (i.e., multinucleated morphology, enlarged nucleus and vacuoles, damage to macromolecules, plasma membrane alterations, and high lysosomal activity) that culminate in a cascade of events leading to the development of SASP [9,57,58,59,60,61]. For instance, protein misfolding and misfolded protein accrual are pathological features of neurodegenerative conditions. A vicious circle of altered proteostasis guiding inflammatory responses of glial cells and inflammatory mediators impinging on proteostasis have been reported [55]. The acquisition of a senescent phenotype relies on autocrine/paracrine signaling involving the type I interferon (IFN) response triggered by DNA damage [62] and mitochondrial dysfunction [14]. Indeed, mitochondrial integrity has been indicated as a major checkpoint for preserving genetic, metabolic, and inflammatory homeostasis [63].

Recent studies have demonstrated that of the MQC mechanisms, functional mitophagy limits age-related neurological decline in aged mice [64]. This control over neurodegeneration may occur via blunting inflammation triggered by mitochondrial dysfunction and mtDNA unloading that activates the cyclic GMP–AMP synthase (cGAS) signaling cascade [64]. However, the activation of mitophagy shows differences across tissues. For instance, higher markers of mitophagy were identified in the retina of old mice together with mitochondrial ultrastructural derangements and cytosolic accumulation of mtDNA [64]. These changes were followed by the cGAS-stimulator of interferon response cGAMP interactor 1 (STING1) signaling activation and downstream IFN-stimulated gene transcription [64]. Such findings are in line with results from primary human dermal fibroblasts from aged individuals and indicate that PINK1-mediated mitophagy is activated during aging in response to mitochondrial dysfunction [64].

**Figure 1:**
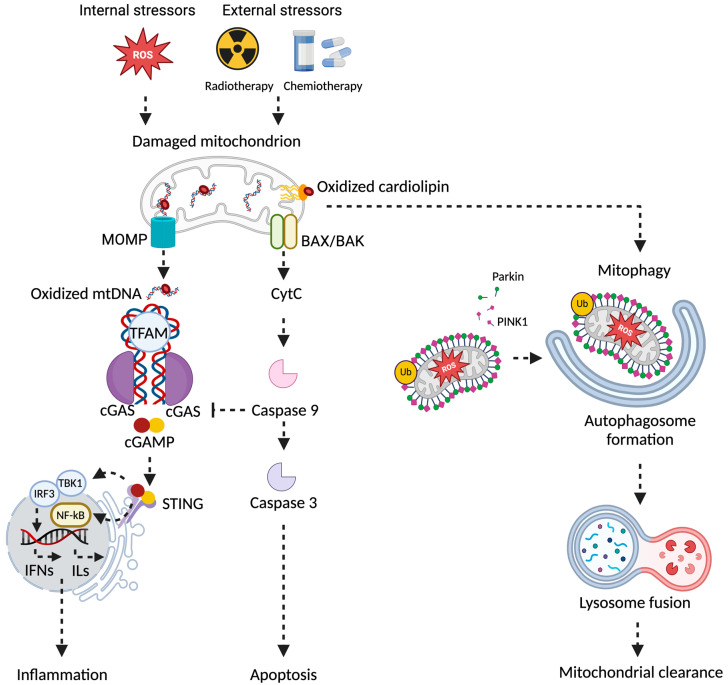
Schematic representation of the central role of mitochondria quality control under stressful conditions and during aging. Mitochondrial dysfunction can result from external (e.g., radiotherapy and chemotherapy) or internal stressors (e.g., reactive oxygen species, ROS). These challenges may lead to mitochondrial membrane permeabilization and mtDNA displacement. The latter can operate as a potent activator of innate immunity via cCGAS- and STING1-dependent signaling, leading to the activation of proinflammatory genes, such as interferon beta 1. An efficient mitochondrial removal by PINK1- and Parkin-dependent mitophagy blunts the age- and stress-induced inflammatory response. Abbreviations: BAK, BCL2 antagonist/killer 1; BAX, BCL2-associated X; cCGAS, cyclic GMP–AMP synthase; IFNs, interferons; ILs, interleukins; IRF3, interferon regulatory factor 3; MOMP, mitochondrial outer membrane permeabilization; mtDNA, mitochondrial DNA; NF-kB, nuclear factor kappa-light-chain-enhancer of activated B cells; PINK1, PTEN-induced kinase 1; STING1, stimulator of interferon response cGAMP interactor 1; TBK1, TANK-binding kinase 1; Ub, ubiquitin. Created with BioRender.com (accessed on 18 June 2024).

The activation of cGAS has been observed in immortalized retinal pigmented epithelial cells treated with an MOMP inducer (ABT-737) and a caspase blocker (QVD) [65,66,67]. ABT-737 drove the accrual of cytosolic mtDNA via oligomerization of BCL2 associated X, apoptosis regulator (BAX) and BCL2 antagonist/killer 1 (BAK1) [65,66,67]. QVD treatment prevented the suppression of cGAS signaling downstream of MOMP [65,66,67]. cGAS activation was abolished by the administration of the mitophagy stimulator urolithin A, which also limited reactive oxygen species (ROS) production and restored mitochondrial oxidative phosphorylation. Finally, the cGAS inhibitor G140 limited MOMP-driven mitophagy in retinal cells but did not blunt mtDNA accumulation in the cytosol [64]. The abrogation of mitophagy by *PINK1* and *PRKN* co-silencing in cells exposed to ABT-737 and QVD exacerbated the MOMP-mediated accrual of cytosolic mtDNA and abolished the beneficial effects of urolithin A administration [68]. Conversely, the inhibition of mitochondrial biogenesis by chloramphenicol reduced the accumulation of mtDNA in the cytosol of ABT-737- and QVD-treated cells while preserving their sensitivity to urolithin A [64]. Altogether, these findings indicate that the modulation of mitophagy in the setting of age-related mitochondrial dysfunction may be, at least partly, under the control of cGAS signaling. Furthermore, a sublethal activation of MOMP has been shown in aged cells, which was still compatible with cell survival but triggered senescence and inflammation [14,69]. Therefore, the modulation of MOMP via sublethal activation of executioner caspases can be exploited for regulating cGAS-triggered inflammation in different settings [14,70,71,72,73], making mitophagy a druggable target for therapeutic exploitation (Figure 1).

Although the study of mitophagy modulation has provided insights into the relationship between mitochondrial dysfunction and inflammation, the pathways of cell death also possess proinflammatory properties and may contribute to aging, as discussed in the next section.

## 4. Mitochondrial Roads to Cell Death: Multiple Pathways toward the Same End

**Figure 2:**
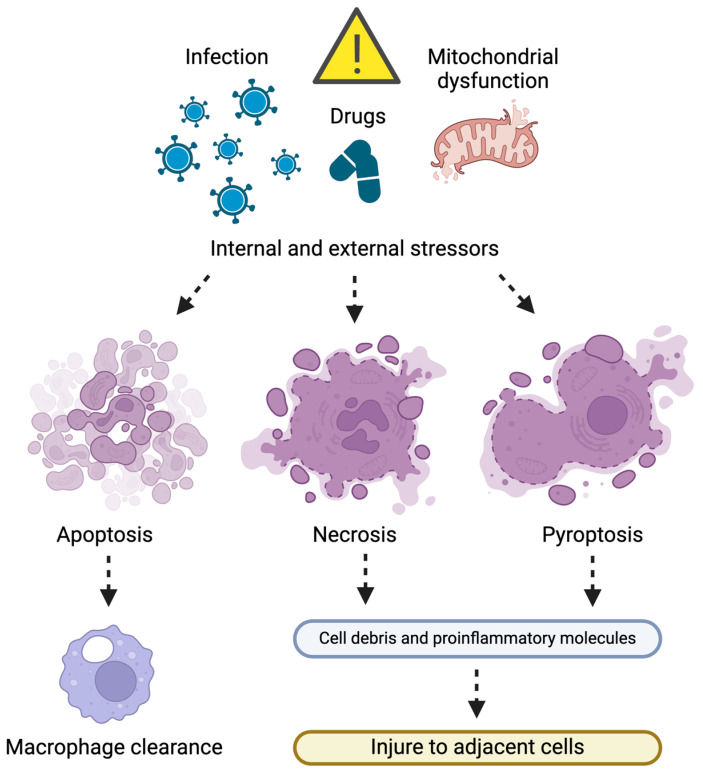
Schematic representation of the main pathways of cell death. Apoptosis generates apoptotic bodies that are cleared by macrophages and is therefore noninflammatory. Instead, necrosis and pyroptosis are proinflammatory cell death pathways that compromise cell membrane integrity and cause cytolysis. In this setting, cell constituents with proinflammatory properties are released, which may act as autoantigens and injure adjacent cells. Created with BioRender.com (accessed on 30 May 2024).

Cell death is the outcome of either apoptosis, a programmed type of cell death, or necrosis, an uncontrolled type of cell death. Alternative forms of programmed cell death have also been described (e.g., pyroptosis), which hold proinflammatory properties but, like apoptosis, follow a series of programmed caspase-dependent events [74] (Figure 2).

### 4.1. Mechanisms of Noninflammatory Cell Death: Apoptosis

Apoptosis is a programmed type of cell death elicited by intrinsic and extrinsic stimuli and executed by cysteine endoprotease (caspase)-dependent and -independent events [75]. Different from necrosis and pyroptosis, apoptosis does not have proinflammatory properties.

The intrinsic pathway of apoptosis is triggered by mitochondrial damage and the release of cytochrome *C* into the cytoplasm. Herein, cytochrome *C* binds to the apoptotic protease-activating factor-1 (Apaf-1) and procaspase-9 and leads to the formation of an adaptor protein complex called the apoptosome [76]. The latter mediates the activation of the apoptotic initiator caspase-9 and the recruitment of caspase-3/7 for cleavage of cellular substrates and ultimately cell death.

The extrinsic apoptotic pathway is triggered upon the binding of pro-cell death signals, such as tumor necrosis factor-α (TNF-α), with death receptors at the cell surface. Following TNF-α binding, the death receptor undergoes oligomerization and recruits caspase-8, which cleaves and activates the apoptotic effector caspase-3 [77,78]. Caspase-8 also cleaves the BH3 domain only pro-apoptotic protein BID to produce a truncated fragment (tBID). The latter migrates to the mitochondrion, where it triggers the integration of BAX at the outer membrane. This event allows formation of BAX/BAK pores on the mitochondrial surface and favors cytochrome *C* release for the activation of the intrinsic apoptotic pathway.

The activation of caspase-3 is a converging point of both intrinsic and extrinsic pathways and an executionary apoptotic component [79,80]. As a result of the caspase-dependent activation of cell death, several biochemical and ultrastructural changes occur, including chromatin condensation, DNA cleavage, membrane blebbing, and flipping of the phospholipid phosphatidylserine at the extracellular side of the plasma membrane for signaling caspase-3 activation [81].

### 4.2. Mechanisms of Proinflammatory Cell Death: Necrosis and Pyroptosis

Necrosis is a proinflammatory uncontrolled form of cell death induced by external stimuli (e.g., hypoxia, inflammation, traumatic injury) and characterized by an increase in cell volume, mitochondrial swelling, and ultimately cellular collapse [82,83]. This process is triggered by a set of cellular changes, including loss of plasma membrane selectivity, ATP depletion, calcium dyshomeostasis, and increased ROS generation, which inflict oxidative damage to cell macromolecules (lipids, nucleic acids, and proteins) [84,85], ultimately leading to the destruction of the cell and organellar membranes [86].

Organelle-mediated necrosis is guided by mitochondrial depolarization and loss of the proton gradient, which trigger mitochondrial swelling. The rupture of the organelle membrane follows the assembly of the mitochondrial permeability transition pore (MPTP) composed of cyclophilin D, a peptidyl-prolyl cis-trans isomerase of the mitochondrial matrix, the voltage-dependent anion channel (VDAC), and the adenine nuclear translocator (ANT) [87,88,89].

The breach of the cell membrane allows for the leakage of proinflammatory mediators into the surrounding environment. This event triggers an inflammatory reaction and tissue injury via nuclear factor kappa B (NF-κB) signaling. Being an energy-independent process, necrosis occurs in cells that are severely damaged by a sudden shock, such as radiation, heat, chemicals, and hypoxia, and become unable to function.

Necrosis can also be triggered via a death receptor pathway elicited by the Fas ligand, TNF-α, and TNF-related apoptosis-inducing ligand binding. The binding of TNF-α to the TNF receptor 1 (TNFR1) is pivotal for exposing the TNFR1 death domain to the cytoplasm and recruiting the TNF-receptor-associated death domain (TRADD) at the plasma membrane. This event guides the formation of the pro-necrosis complex I composed of TNFR1, TRADD, receptor-interacting protein 1 (RIP1), TNF-related apoptosis-inducing ligand receptors (TRAILs), and cellular inhibitors of apoptosis 1/2 (cIAP1/2). Complex I activates NF-κB signaling while blunting caspase activation. Furthermore, the formation and phosphorylation of RIP1/RIP3 complexes leads to the generation of necrotic bodies and activates a set of catabolic enzymes that lead to increased oxidative phosphorylation and ROS production, ultimately triggering necrosis. RIP1/RIP3 phosphorylation also triggers mixed-lineage kinase domain-like protein (MLKL) activation [90,91], which guides mitochondrial disarrangement, altered cell permeability, and finally, necrosis [92].

Pyroptosis is a caspase-dependent apoptotic cell death that operates through proinflammatory signaling [93,94]. Classical and nonclassical pathways of pyroptosis have been described that culminate in the cleavage of gasdermin D (GSDMD) under specific caspase activation cascades. Caspase-1 and caspase-4, caspase-5, and caspase-11 are activated for classical and nonclassical pyroptosis pathways, respectively [93,95]. Following caspase cleavage, the GSDMD C-terminal autoinhibitory domain is removed and the GSDMD N-terminal domain mediates the generation of a large pore in the plasma membrane. This event promotes cell swelling and membrane blebbing and ruptures, which facilitate the release of cell content and ultimately cell death [93,96].

Pyroptosis can also be activated by GSDME cleavage under the activity of executive-apoptotic protein caspase-3 [97,98]. The caspase 3-mediated cleavage of GSDME has been described as a molecular “switch” between apoptosis and pyroptosis in cancer, with higher expression of caspase-3 triggering pyroptosis [97,99].

The caspase 3-guided cleavage of GSDME degrades an autoinhibitory structure and generates a fragment with an active N-terminal domain (GSDME-N). GSDME-N permeates the plasma and mitochondrial membranes, with a preference for the latter, and by inducing ruptures [75], shifts noninflammatory apoptosis into the fast proinflammatory pyroptosis [97,98]. The coordinated rupture of the mitochondrial membrane and pore formation guided by GSDME allows for the release of mtDNA that can act as DAMP and trigger inflammation [100].

Both GSDMD- and GSDME-driven pyroptotic cell death pathways converge on a sterile inflammatory response mounted by the recognition of DAMPs and the release of specific proinflammatory cytokines (i.e., interleukin (IL)-1β and IL-18) [93,101]. The recognition of DAMPs by inflammasome complexes activates proinflammatory processes that assist in the production of IL-1β and IL-18. IL-1β is synthesized by activated macrophages, monocytes, and a subset of dentritic cells as a precursor that is activated by a proteolytic cleavage by caspase 1 and triggers inflammation [102]. IL-18 is also produced as a precursor that is cleaved by caspase-1 for its activation and stimulation of IFN γ signaling and regulation of T helper 1 (Th1) and Th2 cell-mediated responses [75]. In particular, the activation of the NLR family pyrin domain-containing 3 (NLRP3) inflammasome has been involved in several age-related conditions, including type 2 diabetes mellitus and Alzheimer’s disease (AD) [103].

## 5. The Contribution of Pyroptosis to Age-Related Diseases

Pyroptosis-programmed cell death, while having a crucial role in embryonic development, has been implicated in the pathogenesis of several age-related conditions, including AD, cancer, osteoarthritis, and presbycusis [15]. Programmed cell death pathways enacting pyroptosis are at the crossroads between mitochondrial dysfunction and sterile inflammation and are increasingly recognized as biological routes that can be exploited for therapeutic gain.

Inflammasome-mediated inflammation and subsequent pyroptosis have been implicated in neurodegeneration [104]. In particular, a role has been envisioned in the pathogenesis of AD through favoring the spread of the Aβ protein, a major constituent of amyloid plaques, within the brain. A high expression of NLRP1 and NLRP3 inflammasomes has been described in the brain of patients with AD and increased levels of circulating GSDMD have also been reported [105]. Aβ accumulation in neurons seems to support GSDMD cleavage and induce classical pyroptosis via NLRP3-caspase-1 signaling [106]. Aβ-mediated pyroptosis can also be triggered via the expression of high levels of NLRP1 in cortical neurons and caspase-1 signaling [107]. Co-activation of NLRP1 and NLRP3 inflammasomes can also occur as a response to Aβ-mediated aggregation and neurotoxicity, thus eliciting pyroptosis to instigate neuroinflammation via the release of proinflammatory cytokines [107].

Cell death and cytokine release have also been indicated as contributors to the pathogenesis of osteoarthritis. Chronic low-grade inflammation is a major trigger of joint degeneration, with chondrocytes showing ultrastructural changes consistent with pyroptosis-mediated signaling [108]. Furthermore, high levels of pyroptosis-related cytokines (IL-1β, IL-18), produced by fibroblast-like synovial cells, have been found in the synovial fluid of patients with osteoarthritis, together with an increased expression of NLRP1 and NLRP3 inflammasomes [109]. In such a context, the lipopolysaccharide/ATP-mediated activation of caspase-1 and the subsequent cleavage of GSDMD have been identified as pyroptosis signaling routes [109]. Together with pyroptosis-associated inflammatory mediators, a set of DAMPs with proinflammatory properties have been retrieved in the synovial fluid and associated with synovitis. In particular, NLRP1 and NLRP3 inflammasomes and caspase-dependent pyroptosis in fibroblast-like synovial cells trigger the release of the high-mobility group box 1 (HMGB1) protein, which supports cartilage degradation and fuels the release of the proinflammatory cytokines that aggravate osteoarthritis [110]. Following the release of DAMPs, synovial macrophages can activate NLRP3 inflammasome and caspase-1 signaling and initiate classical pyroptosis via the release of IL-1β and IL-18 from the chondrocytes, thus inducing an inflammatory cascade that instigates chondrocyte pyroptosis [111]. High levels of hypoxia-inducible factor-1α (HIF-1α) have also been found in the serum, synovial fluid, and articular cartilage of patients with osteoarthritis. HIF-1α can trigger the NLRP3 inflammasome, activate caspase-1, and elicit classical pyroptosis [112]. Finally, uric acid, an endogenous danger molecule, can ignite the NLRP3 inflammasome and caspase activation, which in turn drives the chondrocyte production of IL-1β and IL-18 [113]. These cytokines can induce chondrocytes to overproduce nitric oxide by upregulating aggrecanases and matrix metalloproteinases. This can result in mitochondrial dysfunction and energy depletion, which can blunt the synthesis and breakdown of the hyaline cartilage matrix [114]. In addition, IL-1β and IL-18 can stimulate chondrocytes and synoviocytes to produce additional inflammatory mediators (e.g., prostaglandin E2) to support synovitis and bone resorption [115]. Joint dysfunction will result from the synergistic effects of these processes, which eventually accelerates the development of osteoarthritis.

Mutations in *DFNA5* (GSDME) have been described in many tissues and organs, especially in the cochlear tissue [116,117]. Gain-of-function mutations lead to truncation of the GSDME protein and progressive sensorineural hearing loss [116,117]. Both GSDME signaling via cytochrome *C* release and caspase-3-dependent pathways and classical caspase-1 signaling have been involved in presbycusis. The generation of mitochondrial ROS during aging, while inflicting per se a damage to the auditory system, contributes to the aggregation of pro-caspase-1, apoptosis-associated speck-like protein containing a CARD (ASC), and NLRP3 inflammasome complex in the cochlea. This triggers inflammation via caspase-1 activation and increased expression of IL-1β and IL-18 [118]. These events mediate cleavage of the GSDMD interdomain and subsequent release of its N-terminus that can perforate plasma membrane and trigger cochlear pyroptosis.

An escape from programmed death is a prominent feature of cancer [119,120] and cells challenged by inflammatory pyroptosis may develop malignant phenotypes [121]. Recently, the activation of pyroptotic genes has been involved in the modulation of tumor immunity and altered MQC and inflammation have been involved in the development and progression of hepatocarcinoma [122,123]. However, the relationship among pyroptosis, mitochondrial dysfunction, and sterile inflammation in cancer is yet to be fully deciphered [123]. Taken as a whole, while altered expressions of pro-pyroptotic bystanders have been found in age-associated conditions, their molecular regulatory network and relationship with mitochondria and sterile inflammation are complex and warrant investigation.

## 6. Conclusions

MQC processes are crucial for controlling cell senescence and death. While programmed inflammatory and noninflammatory cell death have been well characterized, additional routes have been identified that allow the release of cellular constituents via uncanonical routes and trigger inflammatory responses. For instance, the release of mtDNA via MOMP under apoptotic stress has recently been shown to trigger senescence programs. Furthermore, pyroptosis, a caspase-dependent inducer of systemic inflammation that is also elicited by mtDNA release, can contribute to age-related sterile inflammation. However, the exact contribution and interrelationship among mitochondrial signaling, sterile inflammation, pyroptosis, and senescence in the development of age-related conditions are currently unclear and warrant investigation. The complexity and highly intricate set of mitochondrial functions, together with the heterogeneity and low reliability of the available methodological approaches, hampers a clear understanding of these connections. Furthermore, in vitro studies that involve inhibition of mitochondrial responses impose several constraints on the overall cell systems, which require ad hoc validation of the results before drawing conclusions on the molecular effects triggered by stressors. Experimental stressors that are mitochondrial-specific and reversible should be preferred over irreversible and “extreme” manipulations (e.g., mtDNA depletion, dissipation of membrane potential). Indeed, these do not reflect physiologically relevant signaling and likely trigger cell death, thereby impeding the appraisal of response types and downstream events. This knowledge is pivotal for developing therapeutic strategies to alleviate the burden of cell damage and achieve a healthy longevity.
